# Corticosteroids for severe sepsis: an evidence-based guide for physicians

**DOI:** 10.1186/2110-5820-1-7

**Published:** 2011-04-13

**Authors:** Djillali Annane

**Affiliations:** 1 General Intensive Care Unit, Raymond Poincaré Hospital (AP-HP), University of Versailles SQY, 104 boulevard Raymond Poincaré, 92380 Garches, France

## Abstract

Septic shock is characterized by uncontrolled systemic inflammation that contributes to the progression of organ failures and eventually death. There is now ample evidence that the inability of the host to mount an appropriate hypothalamic-pituitary and adrenal axis response plays a major in overwhelming systemic inflammation during infections. Proinflammatory mediators released in the inflamed sites oppose to the anti-inflammatory response, an effect that may be reversed by exogenous corticosteroids. With sepsis, via nongenomic and genomic effects, corticosteroids restore cardiovascular homeostasis, terminate systemic and tissue inflammation, restore organ function, and prevent death. These effects of corticosteroids have been consistently found in animal studies and in most recent frequentist and Bayesian meta-analyses. Corticosteroids should be initiated only in patients with sepsis who require 0.5 μg/kg per minute or more of norepinephrine and should be continued for 5 to 7 days except in patients with poor hemodynamic response after 2 days of corticosteroids and with a cortisol increment of more than 250 nmol/L after a standard adrenocorticotropin hormone (ACTH) test. Hydrocortisone should be given at a daily dose of 200 mg and preferably combined to enteral fludrocortisone at a dose of 50 μg. Blood glucose levels should be kept below 150 mg/dL.

## Introduction

More than half a century after the first randomized, controlled trial on corticosteroids for severe infection [1], there is a broad use of corticosteroids by physicians worldwide despite an amazing contradiction between experts on their benefit-to-risk ratio. Among the various factors that influence physician's practice, one of the most important is the positive or negative physician's own experience with a drug. The broad and longstanding adoption of corticosteroids to treat severe infections likely relies on the prompt reversal often seen at the bedside of life-threatening complications, such as shock and respiratory failure. The current controversy among experts on this topic is not fuelled by new scientific data but rather by distorting differently the reality. This current review was designed to provide readers with a clear and fair evaluation of the rationale for using corticosteroids and with evidence-based decision tree for whom to treat, when, and how.

## Rationale for using corticosteroids

### Evidence for overactivity of proinflammatory pathways relative to endogenous glucocorticoids activity

It is generally accepted that uncontrolled systemic inflammation is the hallmark of severe sepsis and the main contributor for the progression of organ dysfunction and death [2]. Host control of inflammation involves a complex interaction between the neuroendocrine and the immune system [3,4]. At the cellular level, two very bioactive systems tightly interact to maintain homeostasis. The nuclear factor kappa B (NF-κB) system promotes the release of proinflammatory mediators, whereas the glucocorticoid-glucocorticoid receptor alpha (G-GRα) complex inhibits inflammation [5]. These two systems are found in all cells and, at rest, are inactivated; NF-κB by its natural inhibitor inhibitory factor kappa B (I-κB) and the G-GRα complex by a shift from the isoform alpha to the isoform beta. As long as these two systems are equally counteracting each other, homeostasis is preserved. Any imbalance favoring NF-κB bioactivity will results in uncontrolled inflammation. There is ample evidence that such an imbalance may occur in conditions of sustained stress. It was shown that severe surgical stress, such as thoracoabdominal surgery, resulted in postoperative relative adrenal insufficiency and subsequent high circulating levels of proinflammatory mediators and development of organ dysfunction [6]. Very similar findings have been reported for trauma patients, after cardiac or liver surgery [7-9]. The so-called relative adrenal insufficiency contributes to progression of critical illness and eventually to death in both children and adults [10,11]. More specifically, in patients with persistent acute respiratory distress syndrome [12] or septic shock [13], overactivity of NF-κB relative to the G-GRα complex contributes damaging cells, tissues, and organs. The mechanisms behind critical illness-induced adrenal insufficiency have been detailed elsewhere [14]. Briefly, apart from drugs altering cortisol metabolism and anatomical damages to the hypothalamic pituitary adrenal axis, cytokine-induced overactivity of the inducible isoform of nitric oxide synthase (iNOS) triggered neuroendocrine cells apoptosis and caused ACTH and glucocorticoid resistance. Critically ill survivors commonly resumed a normal endocrine function weeks to months after hospital discharge.

### Glucocorticoids molecular mechanisms of action perfectly fit to the pathomechanism of sepsis

Glucocorticoids exert their effects through nongenomic and genomic effects. During the few minutes after exposure to glucocorticoids occur the nongenomic effects, including decreased platelets aggregation, cell adhesions and intracellular phosphotyrosine kinases, and increased annexin 1 externalization [15,16]. These effects likely result from interaction of the glucocorticoid and specific membrane sites [17]. The genomic effects are commonly described as transrepression and transactivation effects [18]. The transrepression effects are considered indirect genomic effects. Indeed, they occur during the few hours after exposure to a glucocorticoid and result from physical interaction between the monomeric G-GRα complexes and NF-κB and AP-1. Then, the nuclear transcription factors are sequestrated in the cytosol and cannot enter the nucleus, preventing the reading of genes encoding for most if not all proinflammatory mediators. The transactivation effects are seen as direct genomic effects. They require a few days of exposure to a glucocorticoid. Indeed, conformational changes are needed with dimerization of the G-GRα complex, which then can enter to the nucleus and interact with glucocorticoid-responsive elements part of genes encoding for regulators of termination of inflammation. Subsequently, key anti-inflammatory factors are upregulated, including phagocytosis, chemokinesis, and antioxidative processes. It is now commonly accepted that the net effect of glucocorticoids on immune cells is reprogrammation rather than inhibition [19]. Indeed, studies using microarray tools demonstrated that there are much more upregulated than downregulated genes after exposure to a glucocorticoid. More recent works have confirmed that glucocorticoids induce specific activated, anti-inflammatory monocytes subtypes that migrate quickly to the inflamed tissues [20]. Glucocorticoids prolonged the survival of this subtype of monocytes via an A3 adenosine receptor triggered anti-apoptotic effects [21]. Obviously, these molecular mechanisms of action of glucocorticoids are very well appropriate to counteract the uncontrolled inflammation that characterized sepsis.

### Glucocorticoids restore cardiovascular homeostasis in sepsis

The mechanisms behind the cardiovascular effects of corticosteroids are not well understood. Corticosteroids induce sodium retention via both mineralocorticoid and glucocorticoid receptors. Thereby, corticosteroids will contribute to correct the hypovolemia that characterizes the early phase of sepsis. In addition, by favoring sodium and water accumulation in blood vessels' wall, corticosteroid will contribute to increase systemic vascular resistance. As detailed elsewhere [22], corticosteroids restore within minutes to hours via nongenomic effects vessels sensitivity to alpha agonist with subsequent increase in mean arterial pressure and systemic vascular resistance. They may interfere with activation of ATP-dependent K+ channel [23]. The enhanced responsiveness to catecholamines is maintained over days via corticosteroids transrepression of genes encoding for iNOS and cyclooxygenase II. The restoration of vascular responsiveness to vasopressor is likely correlated to the intensity of the imbalance between NF-κB and G-GRα complex activity. Nonresponders to 250-μg ACTH test, i.e., having cortisol increment of <250 nmol/L, have a more marked improvement in hemodynamics following intravenous bolus of hydrocortisone [24,25]. There was a very strong correlation between the peak cortisol after ACTH and the peak increase in mean arterial pressure after 50 mg of intravenous hydrocortisone in norepinephrine-treated septic shock [24]. Accordingly, initiation of corticosteroids shortened the duration of vasopressor dependency in septic shock patients, and increased the chance of be weaned off of catecholamines by 35% [26]. Of note, premature interruption of corticosteroids, i.e., stopping treatment after 72 hours may cause rebound in inflammation and worsening of hemodynamic status [27]. Corticosteroids have little if no effect on pulmonary circulation or cardiac index [27]. Treatment with moderate doses of hydrocortisone increased capillary density and perfusion in patients with septic shock [28]. These favorable effects on the microcirculation occurred within 1 hour after hydrocortisone administration and may likely result from upregulation of the endothelial isoform of the nitric oxide synthase via a mitogen-activated protein kinase/Akt-dependent pathway [29]. Finally, corticosteroids may favor the restoration of the physiologic fluctuations in the cardiovascular system [30].

### Corticosteroids restore organs function and decrease intensive care unit length of stay of patients with sepsis

Corticosteroids may both prevent organ failure and reduce the intensity and number of organ dysfunction by reducing tissue inflammation and triggering tissue repair and by improving tissue perfusion. For example, in patients with acute respiratory distress syndrome, exogenous administration of corticosteroids completely blocked NF-κB in the lung [12]. In patients with septic shock, glucocorticoids inhibited the release of tumor necrosis factor from vascular tissues and smooth muscles [31]. In addition, treatment with hydrocortisone fully inhibited NF-κB activity in peripheral mononuclear cells by day 5 of treatment [13]. Corticosteroids have been shown to suppress renal iNOS activity after endotoxemia to prevent hypoxic injuries to the cortex, to improve renal oxygen delivery, and finally to restore renal oxygen consumption [32]. Likewise, in patients with septic shock, corticosteroids improved permeability of the glomerular endothelium [33] and normalized free water clearance and renal sodium excretion (Djillali Annane, personal communication). The favorable effects of corticosteroids on organ perfusion also have been shown for the heart [29] and brain [34,35]. A meta-analysis of five randomized trials demonstrated a strong reduction in the SOFA score at 1 week after randomization (weighted mean difference, -1.47 [-2.01, -0.92], *P *< 0.00001, with no heterogeneity in the results: I^2 ^= 2%) [36]. Corticosteroids favorably affected cardiovascular, lung, liver, and renal functions. Because patients treated with corticosteroids are rapidly weaned off vasopressor therapy and mechanical ventilation, they are discharged much earlier from the intensive care unit. Indeed, In a meta-analysis of eight septic shock trials, corticosteroids decreased by 4.5 days on average intensive care unit length of stay (weighted mean difference: -4.49: -7.04 to -1.94, *P *= 0.00055; and I^2 ^= 0%).

### Corticosteroids and survival from sepsis

Whereas corticosteroids invariably improved survival in endotoxinic or septic animals, clinical studies have reported conflicting results. Nevertheless, recent critical analyses of the available randomized trials have suggested in both frequentist [26,36,37] and Bayesian [38] approaches that low-to-moderate doses of corticosteroids improved survival, whereas a short course with high-dose corticosteroids had no or harmful effects. In the two frequentist meta-analyses, this treatment was associated with a risk ratio (RR) for 28-day mortality of 0.84 (n = 12 trials, I^2 ^= 15%; 95% confidence interval (CI), 0.72-0.97; *P *= 0.02) [26] and an odds ratio (OR) for death of 0.64 (n = 12, I^2 ^= 25%; 95% CI, 0.45-0.93; *P *= 0.02) [37]. In the Bayesian meta-analysis, the authors computed the probability of OR for death as >1 [38]. They included nine trials of low-to-moderate doses of corticosteroids (<1,000 mg per day of hydrocortisone or equivalent). The mortality probability (i.e., that OR was >1 with corticosteroids) was 20.4%. There was strong heterogeneity in the results due to inclusion of one old trial with poor methodological quality, including both children and adults and with a short course (<3 days) of hydrocortisone [39]. When excluding this outlier, the mortality probability was only 5.8%, suggestive of survival benefit from low-to-moderate doses of corticosteroids. Because there is mega-trial of corticosteroids for sepsis, the best evidence is the one provided by high-quality systematic reviews and meta-analysis, suggesting survival benefit from prolonged treatment with low-to-moderate dose of corticosteroids.

### Corticosteroids safety in sepsis

The recent meta-analyses of corticosteroids for severe sepsis consistently did not find any increase in the risk of superinfection, gastroduodenal bleeding, or muscle weakness [2,36-38]. There often is a misunderstanding of the findings from CORTICUS [40]. In CORTICUS, hydrocortisone was not associated with an increase in the rate of infection in the lung, abdominal, urinary tract, wound tissues, or the rate of catheter-related infection of primary septicemia. Hydrocortisone-treated patients had a higher rate of shock relapse, which may not necessarily be related to documented new infections.

## Use of Corticosteroids in practice: the "Who," "When," and "How"

### Optimal target population for corticosteroids

In this review, we will not consider the use of corticosteroids in specific infections, such as bacterial or tuberculosis meningitis, severe typhoid fever, or *Pneumocystis carinii *infections in the immune-compromised patient.

**Figure 1:**
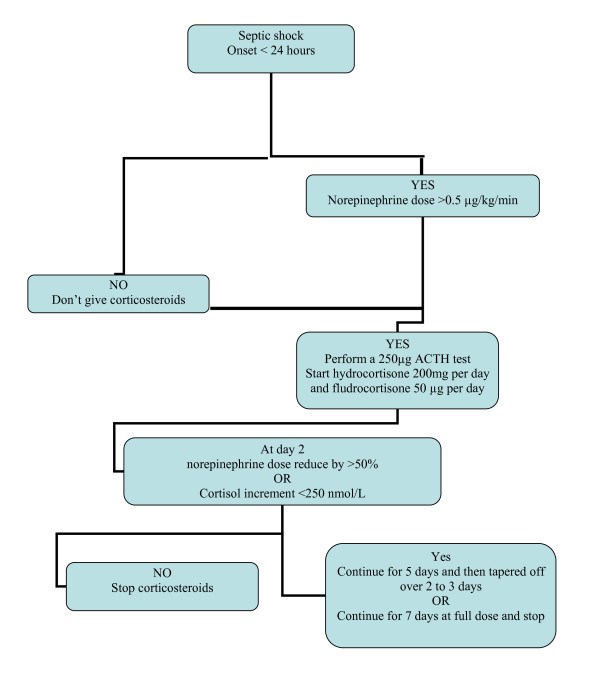
**Decision tree for practical use of corticosteroids at bedside**.

A necessary condition to initiate corticosteroids in patients with severe infections is the need for vasopressor therapy. Moreover, the recent meta-analyses found a strong and negative correlation between the severity of sepsis and the relative risk of dying [36-38]. The meta-regression analysis suggested that low-to-moderate doses of corticosteroids are more likely to improve survival in patients with a baseline risk of death of 44% or more. One of the major differences between the French Ger-Inf-05 study [25] and CORTICUS [40] was baseline severity of septic shock. More specifically, in the former trial patients had to require dose of vasopressors that were roughly twice greater than in CORTICUS (on average 1.1 μg/kg/min vs. 0.5 μg/kg/min of norepinephrine). Of note, analysis of the subgroup of CORTICUS patients (n = 126) who met the same entry criteria than those requested in the French Ger-Inf-05 trial found survival benefits that were very consistent with findings of the French trial. Indeed, 63 of 126 patients died at 28 days after randomization. There were 31 of 69 (45%) deaths in the hydrocortisone-treated group and 32 of 57 (56%) in the placebo group, corresponding to a -11% absolute reduction in 28-day mortality. In practice, corticosteroids should be initiated in patients with sepsis requiring >0.5 μg/kg/min of norepinephrine or equivalent (Figure 1).

None of the recent meta-analysis found differences in treatment benefits in nonresponders versus responders to the ACTH test [3-38]. However, in the French Ger-Inf-05 trial [25], only nonresponders to the 250-μg ACTH test (cortisol increment < 250 nmol/L) drew benefit from corticosteroids. Whereas in CORTICUS primary analysis, there was no interaction between treatment effects and the results of the ACTH test [40], in the subpopulation mimicking the French trial population, hydrocortisone significantly decreased mortality in the nonresponders (hazards ratio (HR) = 0.45; 95% CI, 0.21-0.93) and had no effect in the responders (HR = 0.9; 95% CI, 0.45-1.78). Thus, it is this author's opinion that an ACTH test should be performed when initiating corticosteroids. The results of diagnostic tests should be taken into account for the decision to stop or continue treatment (see below).

### When corticosteroids should be initiated? And stopped?

Animal experiments have demonstrated that baboons challenged with a lethal dose of endotoxin had a greater survival chance when corticosteroids were initiated within the first 4 hours, even though delayed treatment was associated with better survival rates than controls [41]. Five randomized trials investigated the effects of low-to-moderate doses of corticosteroids when initiated within the 24 hours of onset of severe sepsis [25,42-46]. There were 118 deaths among the 222 corticosteroids-treated patients and 139 deaths among the 223 controls (RR = 0.85; 95% CI, 0.73-0.99; *P *= 0.03). There was no heterogeneity across the studies (I^2 ^= 0%). In contrast, in five studies with a time window of up to 72 hours [40,47-49], corticosteroids had no effect on survival (RR = 0.72; 95% CI, 0.48-1.1; *P *= 0.13) and there was some heterogeneity across the studies (I^2 ^= 49%). Thus, based on findings from animal experiments and clinical trials, corticosteroids should be initiated within the first 24 hours of septic shock.

Recommendations from experts suggest that treatment with corticosteroids should be weaned off over 3 to 6 days after 5 days of treatment at full dose [50,51]. Due to the genomic nature of sustained corticosteroids effects, treatment should be prolonged for several days. Meta-regression in a recent meta-analysis suggested that the duration of treatment at full dose is a strong predictor of survival benefit from corticosteroids with the "neutrality line" being crossed by the regression line for time equal to 120 hours arguing for maintaining treatment at full dose for at least 5 days [26,36]. In this meta-analysis, there was no evidence for better survival rates in studies with versus without tapering. It is this author's opinion that corticosteroids should be stopped in patients whose vasopressor dependency has not improved after 2 days of treatment and are responders to the 250-μg ACTH test.

### How should corticosteroids be given?

As highlighted in the first part of this review, the rationale for using corticosteroids in septic shock relies on the concept of critical illness associated corticosteroids insufficiency [51]. Hydrocortisone, the natural hormone, should be preferred to synthetic corticosteroids. The commonly accepted dose is on average 200 mg per day. A recent meta-analysis demonstrated that the lower the corticosteroids dose the greater the response to treatment [26,36]. Hydrocortisone may be given as boluses or as a continuous infusion. Whereas a continuous infusion may be associated with less glucose variability [52], it also may favor adrenal insufficiency after withdrawal of corticosteroids. A recent randomized trial of corticosteroid-treated septic shock found no evidence for a benefit of normalizing blood glucose levels versus maintaining levels <150 mg/dL [53]. The adjunction of fludrocortisone to hydrocortisone remains controversial. In a recent randomized trial, there was a -3% nonstatistically significant absolute reduction in mortality with hydrocortisone plus fludrocortisone versus hydrocortisone alone. However, this trial was not powered for this analysis, there was no fludrocortisone placebo, and the study was not blinded. Thus, due to the fact that the French Ger-Inf-05 trial has tested the combination of hydrocortisone and fludrocortisones, it is still this author's opinion that fludrocortisones should be given via the nasogastric tube at a dose of 50 μg per day.

## Conclusions

There is a strong biological rationale to support the use of low-to-moderate doses of corticosteroids for at least 5 days before tapering. Animal studies and recent frequentists or Bayesian meta-analyses consistently have demonstrated survival benefit with this treatment particularly when given to the sickest patients, i.e., those who require 0.5 μg/kg per minute or more of norepinephrine. Treatment should be initiated within the first 24 hours and should consist of both hydrocortisone and fludrocortisone.

## Competing interests

The author declares that they have no competing interests.
